# An epidemiological study of the risk factors associated with myopia in young adult men in Korea

**DOI:** 10.1038/s41598-017-18926-2

**Published:** 2018-01-11

**Authors:** Dong Cheol Lee, Se Youp Lee, Yu Cheol Kim

**Affiliations:** Department of Ophthalmology, Dongsan Medical Center, Keimyung University School of Medicine, Daegu, 41931 Korea

## Abstract

The prevalence of myopia has been increasing worldwide. Its causes are not completely clear, although genetic and environmental factors are thought to play a role. Data were collected by the Korean Military Manpower Administration. Frequency analysis was used for comparisons of general characteristics. Pearson’s chi-square tests and logistic regression analysis were used to verify the correlations between possible risk factors and the prevalence of myopia or high myopia. The prevalence of myopia (50.6–53.0%) and high myopia (11.3–12.9%) increased each year. These tended to be the highest in patients born in spring, and decreased in the following order according to education level: 4- or 6-year university education or more, high school education or less, and 2- to 3-year college education. Moreover, the prevalence of myopia and high myopia was significantly higher in patients ≤ 60 kg and with a body mass index ≤ 18.5 kg/m^2^. The prevalence of high myopia was significantly higher in taller patients (≥175 cm). The prevalence of myopia and high myopia increased each year in Korean young adult men and was associated with birth season, education level, height, weight, and body mass index. Tall, lean men were more likely to have high myopia.

## Introduction

The prevalence of myopia has been increasing worldwide^1^. Although its causes are not completely clear, genetic and environmental factors may play a role; the contributions of environmental factors are relatively high^2^. Key indicators of the genetic basis of myopia include familial clustering^3^, ethnic background^2,4–16^, and twin and familial correlation studies^17^. Environmental risk factors for myopia include education and intelligence^18,19^, near work^7,20^, urbanization^12,13^, prenatal factors (premature birth and low birth weight)^21^, socioeconomic status^5,14^, body stature (height, weight), body mass index (BMI)^19,22–28^, malnutrition^2^, birth season^29^, light^30^, and time spent outdoors^31^. However, the association between these factors, and myopia and high myopia is unclear.

Population-based studies with high response rates, sufficient population sizes, and few biases provide robust evidence for determining the aetiology of myopia. Therefore, in this study, we examined the prevalence of myopia and high myopia, and the association of environmental risk factors, such as body stature (height, weight), body mass index (BMI), education level, and birth season, using a survey of young adult Korean men who underwent physical examinations from 2009 to 2013 performed by the Korean Military Manpower Administration (MMA).

## Results

### Prevalence of myopia and high myopia in the overall population

In this study, a total of 1,784,619 Korean men between the ages of 18 and 35 years (mostly 19-years-old) were enrolled from 2009 to 2013 (Supplementary Table S1). The prevalence rates of myopia and high myopia ranged from 50.6% to 53.0% and from 11.3% to 12.9%, respectively. From 2011, the prevalence of myopia and high myopia increased (*P* < 0.001; Table 1).

**Table 1:** Prevalence of myopia and high myopia over a 5-year period.

Prevalence	2009	2010	2011	2012	2013	*P*-value**
Myopian (%)	167,037	188,761	187,491	186,650	187,897	<0.001
	(50.8)*	(53.0)*	(50.6)*	(51.3)*	(51.5)*	
High myopian (%)	37,135	42,038	43,376	45,884	47,065	<0.001
	(11.3)*	(11.8)*	(11.7)*	(12.6)*	(12.9)*	

^*^Statistically significant.

^**^Analysed with Pearson’s significance ratio.

### Prevalence of myopia according to environmental risk factors

**Figure 1:**
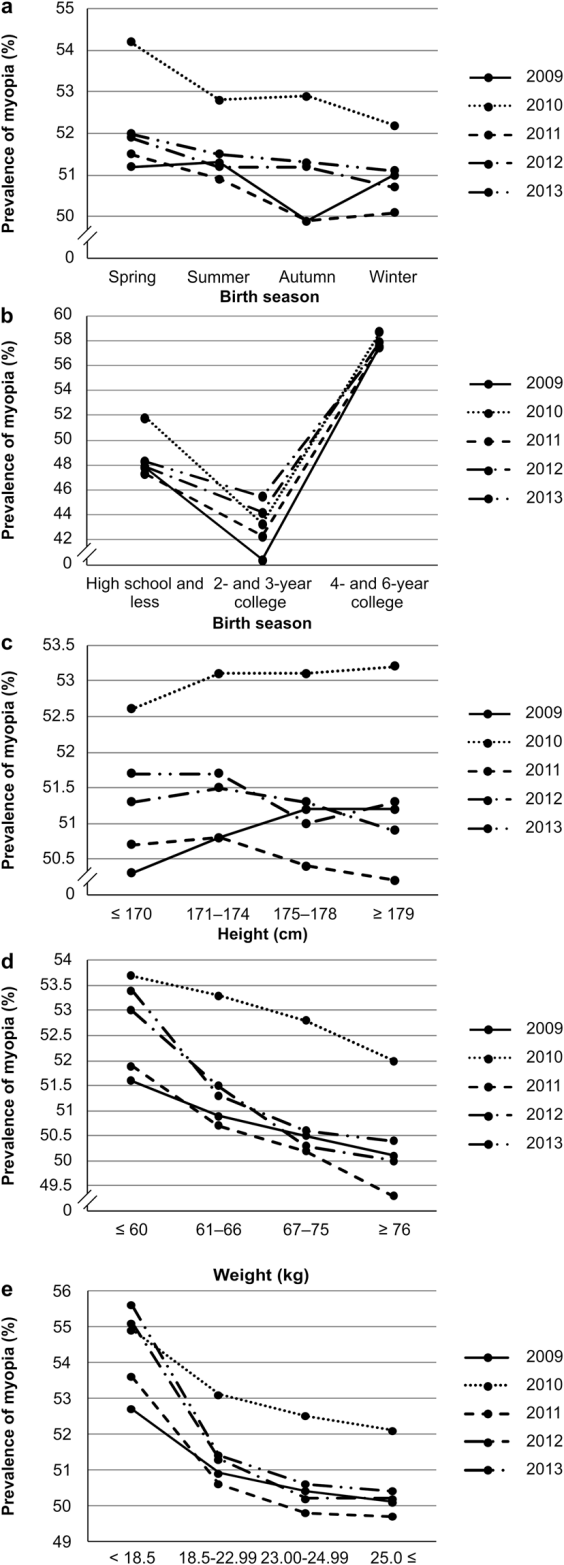
Prevalence of myopia according to (**a**) Birth season. (**b**) Education level. (**c**) Height. (**d**) Weight. (**e**) Body Mass Index (BMI).

Myopia was most prevalent in participants born in spring, except those examined in 2009 (Fig. 1a). In 2009, the prevalence was higher in participants born in spring, summer, and winter than in those born in autumn (*P* < 0.001). In 2011 and 2013, the prevalence was higher in participants born in spring and summer than in those born in other seasons (*P* < 0.001 and *P* < 0.01, respectively; Supplementary Table S2). The results of the multivariable logistic regression analysis with spring as a reference are shown in Supplementary Table S3.

The prevalence of myopia in participants according to level of education was consistent over the 5-year period. Myopia was more prevalent in those with a 4- or 6-year college education or higher, compared to other education levels (*P* < 0.001; Supplementary Table S2, Fig. 1b). Supplementary Table S3 shows the results of the multivariable logistic regression analysis, using participants with a high school education or less as the reference.

The prevalence of myopia had no consistent relationship with height (Supplementary Table S2, Fig. 1c). In 2009, the prevalence of myopia was higher in participants ≥175 cm (*P* < 0.01). In 2010, it was higher in participants ≥171 cm (*P* < 0.05). For 2011 and 2013, it was higher in participants ≥175 cm (*P* < 0.05 and *P* < 0.01, respectively). The results of the multivariable logistic regression analysis using participants <170 cm as the reference are shown in Supplementary Table S3.

The prevalence of myopia according to weight increased from 2009 to 2012, with participants <66 kg showing a higher prevalence than those weighing ≥67 kg. In 2013, the prevalence was higher in participants <60 kg than in those ≥61 kg (*P* < 0.001). Prevalence tended to increase as weight decreased (Supplementary Table S2, Fig. 1d). Supplementary Table S3 shows the results of the multivariable logistic regression analysis using participants <60 kg as a reference.

The prevalence of myopia according to BMI was consistent across the 4-year period from 2009 to 2012, and higher in participants with a BMI < 23.0 kg/m^2^ than in participants with a BMI ≥23.0 kg/m^2^. In 2013, the prevalence of myopia was higher in participants with a BMI <18.5 kg/m^2^ than that in other groups (*P* < 0.001). Accordingly, as the BMI decreased, the prevalence of myopia tended to increase (Supplementary Table S2, Fig. 1e). Supplementary Table S3 shows the results of the univariate logistic regression analysis using participants with a BMI <18.5 kg/m^2^ as the reference.

### Prevalence of high myopia according to environmental risk factors

**Figure 2:**
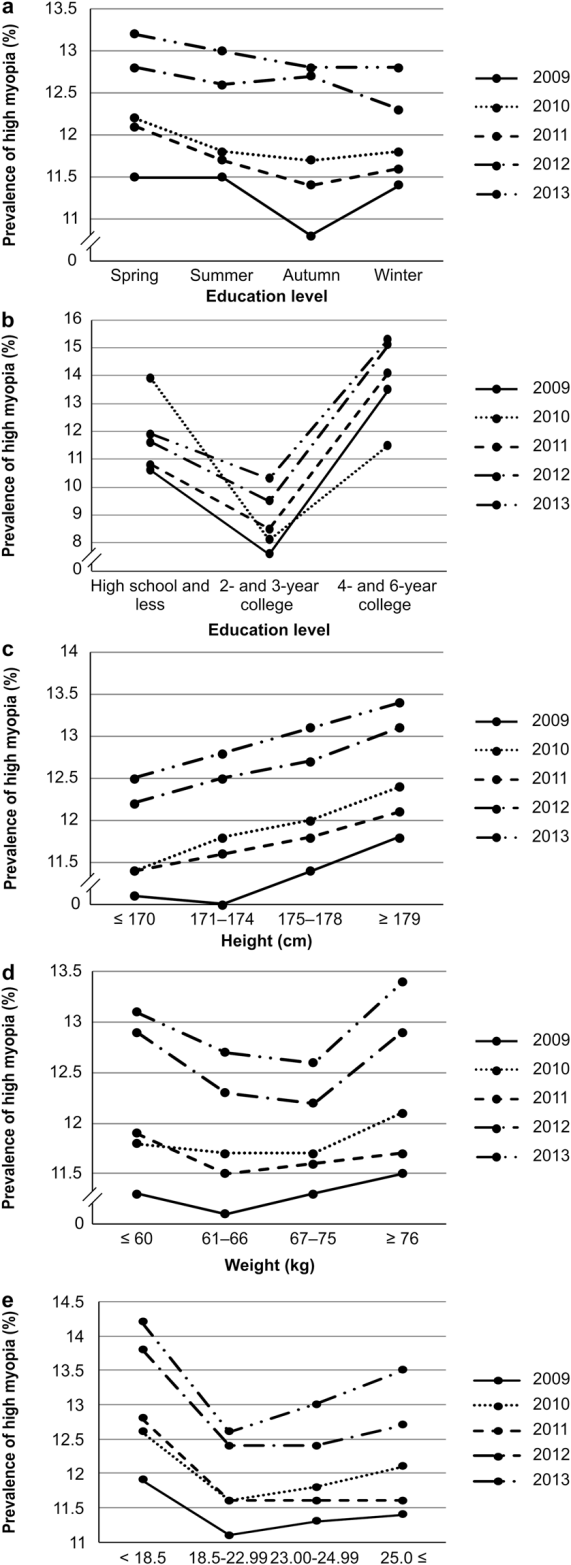
Prevalence of high myopia according to (**a**) Birth season. (**b**) Education level. (**c**) Height. (**d**) Weight. (**e**) Body Mass Index (BMI).

The prevalence of high myopia was highest in participants born in spring, except for those participants examined in 2009 (Fig. 2a). In 2009, the prevalence was higher in participants born in spring, summer, and winter than in participants born in autumn (*P* < 0.001). In 2011 and 2013, the prevalence was higher in participants born in spring and summer than in those born in other seasons (*P* < 0.001 and *P* < 0.05, respectively). In 2012, the prevalence was higher in participants born in spring, summer, and autumn than in those born in the winter (*P* < 0.05; Supplementary Table S4). The results of the multivariable logistic regression analysis with spring as the reference are shown in Supplementary Table S5.

The prevalence of high myopia according to level of education was consistent over the 5-year period. Participants with a 4- or 6-year college education or more had a higher prevalence of high myopia than other education levels, except for those examined in 2010 (*P* < 0.001; see Supplementary Table S4, Fig. 2b). The results of the multivariable logistic regression analysis using participants with a high school education or less as the reference are shown in Supplementary Table S5.

The prevalence of high myopia according to height remained consistent over the 5-year period, with participants over 175 cm in height having a higher prevalence of high myopia than participants less than 175 cm in height (*P* < 0.001; see Supplementary Table S4, Fig. 2c). The results of the multivariable logistic regression analysis using participants <170 cm as the reference are shown in Table, Supplementary Table S5. During this period, taller individuals were more likely to have high myopia.

The prevalence of high myopia did not differ according to weight in 2009 and 2010 (*P* > 0.05 for both). From 2011 to 2013, participants weighing <60 kg or >75 kg had a higher prevalence of high myopia than the other participants (*P* < 0.05, *P* < 0.001, and *P* < 0.001, respectively). There were no consistent trends over the 5-year period (Supplementary Table S4, Fig. 2d). Supplementary Table S5 shows the results of the multivariable logistic regression analysis using participants <60 kg as the reference.

In 2009, the prevalence of high myopia according to BMI was higher in participants with a BMI <18.5 kg/m^2^ or >23.0 kg/m^2^, than in participants with a BMI of 18.5–22.99 kg/m^2^. In 2011, the prevalence of high myopia was higher in participants with a BMI <18.5 kg/m^2^ than that in the other groups. In 2010, 2012, and 2013, the prevalence was higher in participants with a BMI <18.5 kg/m^2^ or >25 kg/m^2^ than that in the other groups. Thus, across the 5-year period, participants with a BMI <18.5 kg/m^2^ had the highest prevalence of high myopia (*P* < 0.001; Supplementary Table S4, Fig. 2e). The results of the univariate logistic regression analysis using participants with a BMI <18.5 kg/m^2^ as the reference are shown in Supplementary Table S5.

## Discussion

Myopia is a major cause of visual problems worldwide. The prevalence of myopia varies by country, age, and ethnic group^32^, and has been reported to be high in East Asian countries^4–11,33^, but much lower in India and South Asia^12–16^. In the current study, we have shown that the prevalence of myopia (50.6–53.0%) and high myopia (11.3–12.9%) in Korean men around 19 years of age increased from year to year beginning in 2011, which was lower than in previous reports. This may be because the Korean MMA measures refractive error in both eyes only when the UCVA of either eye was 0.3 or worse. Thus, it is possible that myopic participants with a UCVA better than 0.4 were not considered as having myopia. However, the proportion of high myopia in all participants is expected to be more reliable because it is unlikely that patients with high myopia would have two eyes with UCVA better than 0.3. Additionally, some myopic eyes with anisometropia might have been excluded from the myopic group after averaging the refraction values of both eyes, and many participants may have had refractive surgery before physical examination. In previous reports, the prevalence of myopia in 19-year-old males (n = 23,616) in Seoul (capital of South Korea) was 96.5%. The prevalence of high myopia was 21.61%^19^. The prevalence of myopia is also known to be higher in urban areas^12,13^. This nation-wide dataset (n = 1,784,619) showed a consistent prevalence of myopia and high myopia with high reliability.

Our analysis found that birth season, education level, height, weight, and BMI could be associated with myopia and high myopia. Participants who were born in spring had an increased prevalence of myopia and high myopia compared with those born in other seasons. Previous studies have also reported that birth season is associated with the prevalence of myopia^29,34–36^. In a study of 276,911 Israeli individuals, Mandel *et al*. reported that myopia was associated with birth during summer. This may be related to natural light exposure during the early perinatal period due to abnormal diurnal growth rhythms^34^. McMahon *et al*. reported that the incidence of high myopia is increased in individuals from the United Kingdom born in summer or autumn rather than in winter. However, this was thought to be associated with other factors that vary according to the season, such as birth weight^29^. Vannas *et al*. showed that the prevalence of myopia was higher in northern Finland than in southern Finland because of the extremely long photoperiod during summer^36^. On the other hand, Norton *et al*. reported that natural and high-intensity light prevent eye growth due to the dopamine release mechanism and promote emmetropization by cornea flattening using an animal model. Ambient or nursery light affects refractive error as it leads to a lack of emmetropization. However, from these studies, the specific mechanism through which birth season affects myopia is not known^36^. Notably, across 4 years of the 5-year study, our results consistently showed that the prevalence was higher in participants born in spring. However, owing to the differences between our study and the other studies described above, there are likely other factors affecting the prevalence of myopia, including ethnicity, sex, region, childcare method, seasonal variations, and other environmental factors. Further studies in groups of children at specific ages are needed to determine the association between birth season and the prevalence of myopia.

Education level is known to be associated with the prevalence of myopia^37^. This may be because individuals with a higher education spend more time doing near work activities, which is a known risk factor for the development of myopia^18,20^. Animal models have also suggested that hyperopic retinal image defocus, which is induced by near work, may play a major role in refractive error development and ocular growth in primates^38^ and chicks^39^. Accordingly, we found that participants with higher education levels (4- to 6-year college education admission) had a higher prevalence of myopia. Participants with a high school education or less showed higher rates of myopia than those with 2- to 3-year college education. We suggest two possible reasons for this. First, the group may have included many participants who failed the entrance examinations for a 4- to 6-year college placement. During the period of data collection, they would have been studying to make another attempt at a 4- to 6-year college admission. As a result, a group of high school education participants might have a higher educational level than those with 2- to 3-years of college education in a South Korean education system. On the other hand, during high school education, the population might be spending more time in near sight through the use of smart-phones, computers, and televisions. Although this population had a low educational level, the greater the amount of near-sighted time, the greater the prevalence of myopia and high myopia^7,10^ compared with the 2- to 3-year college education population. In recent years, as the number of near-sighted devices has increased, the probability of developing myopia and high myopia has increased. In particular, when people use smartphones, they behave in the same way as ‘near work’ while attempting to see small-sized texts and videos on the smartphones. It is known that the smartphone became popular in Korea in 2009. Since then, smartphone usage has been increasing every year. The number of smartphone users has increased from 470,000 in November 2009 to more than 33 million in January 2013^40^.

As of May 2012 in Korea, 76% of teens and 93.5% of young adults in their 20’s reportedly have smartphones^41^.

The contribution of height to myopia has been assessed in several population-based studies, especially in young adults^25–27^. Hang *et al*. found a significant association between axial length (AL) and height in Chinese twins^27^. Additionally, Sharma *et al*. reported that height was inversely associated with refractive error (taller children were more myopic) among Chinese boys, but not Chinese girls^25^. In contrast, Rosner *et al*. found no relationship between myopia and body stature in a study of 106,926 Israeli male military recruits aged 17–19 years^26^. These inconsistencies may result from ethnic and demographic differences, and the relationship between myopia and height is still unclear. Our study showed that height was also a risk factor for high myopia, with high myopia being more prevalent in taller participants.

Previous reports have shown that height is associated with AL^23,27^. Changes in AL may involve remodelling of the scleral extracellular matrix, which would increase AL because of the lengthening of the vitreous chamber^42^. In our study, the prevalence of high myopia was positively associated with height, indicating an association with AL. Thus, high myopia may be associated with connective tissue properties, such as sclera composition. Additionally, myopia may be affected by the process of emmetropization, which involves cornea flattening. In myopia, the refractive error induced by AL elongation can be compensated for by cornea flattening. However, in high myopia, the extent of AL cannot be compensated for.

In this study, we found that decreased BMI was associated with an increased prevalence of myopia and high myopia. Wu *et al*. showed that heavier individuals tended to be slightly hyperopic^22^, and Wong *et al*. reported that individuals with a higher BMI were more likely to be hyperopic than lighter, leaner persons^28^. Gunes *et al*. reported that retrobulbar fat is clearly limited by the orbital space, preventing expansion^24^, unlike other fat tissue depots in the body^43^. Therefore, obese individuals tend to have more hyperopic vision and shorter vitreous chambers^28^. However, Jung *et al*. showed that myopic refractive error was not associated with weight or BMI^19^. Therefore, we concluded that weight and BMI were more closely related to emmetropization than AL factors in myopia. Because of the limited orbital space, the eyes of obese men may not grow as well as those of leaner men, as shown by Gunes *et al.*^24^. Overall, our results suggested that young Korean men who were tall and lean may tend to acquire high myopia owing to a long AL. High myopia is also associated with other complications affecting vision, including age-related cataracts, myopic macular degeneration, choroidal neovascularization, and open angle glaucoma^2,44^. Further studies in similar cases may reveal connections between connective tissue diseases and other diseases of vision, facilitating the development of therapies for preventing high myopia.

Although we obtained meaningful results, this study had several limitations. First, some of the participants (3–4%) received physical examinations before or after the age of 19 (age range: 18–35 years). This may have led to some variation in the results. Second, we only included men in this study. Myopia has been reported to be more prevalent in women than in men^45^. Therefore, our data may underestimate the prevalence of myopia and high myopia in the overall population. Third, in general, autorefraction was completed without cycloplegia. In previous studies, when the autorefractor did not use a cycloplegic agent, it tended to measure myopia^46^. Therefore, it might have resulted in a higher myopia prevalence in the present study. However, the age of the current population was above 19 years; therefore, this population is minimally affected by accommodation and the result might be more accurate compared with the findings from children. Additionally, this was a cross-sectional, retrospective study; thus, the specific causes of myopia and associated risk factors could not be determined from the current study.

In conclusion, our results showed that the prevalence of myopia and high myopia increased from year to year in Korean men, and in association with birth during spring and a high education level. Additionally, high myopia was more common in tall, lean men.

## Methods

### Participants

The study design followed the tenets of the Declaration of Helsinki for biomedical research in human subjects. ‘Approval of audit exemption’ was obtained from the appropriate institutional review board. Informed consent was not required for this type of study. The Korean MMA conducts yearly physical examinations to determine suitability for military service. This study was based on data acquired by the MMA from 2009 to 2013. All participants were aged 18–35 years; most were 19-years-old (96–97%).

### Refraction

Refractive errors in both eyes were measured in participants whose uncorrected visual acuity (UCVA) in either eye was worse than 0.3 (by Snellen equivalent visual acuity chart) using an autorefractometer (R-F10; Canon Inc., Tokyo, Japan), generally without cycloplegia. Refraction measurements were converted into spherical equivalents, calculated as the spherical value plus half of the astigmatic value (sphere +0.5 cylinders). The mean spherical equivalents of two eyes were used as statistical data. Myopia was defined as ‘−0.50 D ≥ myopia > −6.0 D’, and high myopia was defined as −6.0 D or worse.

### Height, weight, and BMI

Each individual was instructed to remove heavy clothing and footwear before height and weight measurement using a calibrated electronic machine. BMI was determined as follows: weight (kg)/height (cm)^2^. Each variable was divided into four quartiles (height: ≤170, 171–174, 175–178, and ≥179 cm; weight: <60, 61–66, 67–75, and ≥76 kg). BMI was divided into four groups using obesity criteria (<18.5, 18.5–22.99, 23.00–24.99, and ≥25.0 kg/cm^2^) defined by the World Health Organization (WHO)^47^.

### Education level and birth season

Data regarding birth season and education level were obtained from administrative recruitment documents. Educational level (admission history) was classified into three categories: high school education or less, 2- to 3-year college education, and 4- or 6-year university education or more. Birth season was classified into four categories: spring (March to May), summer (June to August), autumn (September to November), and winter (December to February).

### Statistics

Statistical analyses were conducted using SPSS version 21.0 (SPSS, IBM Corp, Armonk, NY, USA). Pearson’s chi-square tests and logistic regression analyses with the lowest point serving as the reference point were used to verify the correlation between the possible risk factors (education level, birth season, height, weight, and BMI) and the prevalence of myopia and high myopia. The results were reported as odds ratios (ORs) with 95% confidence intervals (CIs). The null hypotheses of no difference were rejected if the *P*-value was less than 0.05.

### Data availability

All data generated or analysed during this study are included in this published article (and its Supplementary Information files). Data on refractive error and possible risk factors were collected by the Korean Military Manpower Administration to determine general features. The authors have the data with essential elements. To obtain this data, one requires approval from an institutional review board.

## Electronic supplementary material

Supplementary Tables

## References

[1] Morgan IG, Ohno-Matsui K, Saw SM (2012). Myopia. Lancet..

[2] Saw SM (1996). Epidemiology of myopia. Epidemiol Rev..

[3] Lam DS (2008). The effect of parental history of myopia on children’s eye size and growth: results of a longitudinal study. Invest Ophthalmol Vis Sci..

[4] Matsumura H, Hirai H (1999). Prevalence of myopia and refractive changes in students from 3 to 17 years of age. Surv Ophthalmol..

[5] Lim HT (2012). Prevalence and associated sociodemographic factors of myopia in Korean children: the 2005 third Korea National Health and Nutrition Examination Survey (KNHANES III). Jpn J Ophthalmol..

[6] Yoon KC (2011). Prevalence of eye diseases in South Korea: data from the Korea National Health and Nutrition Examination Survey 2008–2009. Korean J Ophthalmol..

[7] Saw SM (2002). Near-work activity, night-lights, and myopia in the Singapore-China study. Arch Ophthalmol..

[8] Lin LL, Shih YF, Hsiao CK, Chen CJ (2004). Prevalence of myopia in Taiwanese schoolchildren: 1983 to 2000. Ann Acad Med Singapore..

[9] Lam CS, Lam CH, Cheng SC, Chan LY (2012). Prevalence of myopia among Hong Kong Chinese school children: changes over two decades. Ophthalmic Physiol Opt..

[10] You QS (2014). Prevalence of myopia in school children in greater Beijing: the Beijing Childhood Eye Study. Acta Ophthalmol..

[11] Wu JF (2013). Refractive error, visual acuity and causes of vision loss in children in Shandong, China. The Shandong Children Eye Study. PLoS One..

[12] Dandona R (2002). Refractive error in children in a rural population in India. Invest Ophthalmol Vis Sci..

[13] Murthy GV (2002). Refractive error in children in an urban population in New Delhi. Invest Ophthalmol Vis Sci..

[14] Ghosh S, Mukhopadhyay U, Maji D, Bhaduri G (2012). Visual impairment in urban school children of low-income families in Kolkata, India. Indian J Public Health..

[15] Pokharel GP, Negrel AD, Munoz SR, Ellwein LB (2000). Refractive error study in children: results from Mechi Zone, Nepal. Am J Ophthalmol..

[16] Goh PP, Abgariyah Y, Pokharel GP, Ellwein LB (2005). Refractive error and visual impairment in school-age children in Gombak District, Malaysia. Ophthalmology..

[17] Hammond CJ, Snieder H, Gilbert CE, Spector TD (2001). Genes and environment in refractive error: the twin eye study. Invest Ophthalmol Vis Sci..

[18] Rosner M, Belkin M (1987). Intelligence, education, and myopia in males. Arch Ophthalmol..

[19] Jung SK (2012). Prevalence of myopia and its association with body stature and educational level in 19-year-old male conscripts in seoul, South Korea. Invest Ophthalmol Vis Sci..

[20] Saw SM, Hong CY, Chia KS, Stone RA, Tan D (2001). Nearwork and myopia in young children. Lancet..

[21] Varghese RM, Sreenivas V, Puliyel JM, Varugheses S (2009). Refractive status at birth: Its relation to newborn physical parameters at birth and gestational age. PLoS One..

[22] Wu HM (2007). Association between stature, ocular biometry and refraction in an adult population in rural Myanmar: the Meiktila eye study. Clin Experiment Ophthalmol..

[23] Ojaimi E (2005). Effect of stature and other anthropometric parameters on eye size and refraction in a population-based study of Australian children. Invest Ophthalmol Vis Sci..

[24] Gunes A, Uzun F, Karaca EE, Kalayci M (2015). Evaluation of anterior segment parameters in obesity. Korean J Ophthalmol..

[25] Sharma A (2010). Height, stunting, and refractive error among rural Chinese schoolchildren: the See Well to Learn Well project. Am J Ophthalmol..

[26] Rosner M, Laor A, Belkin M (1995). Myopia and stature: findings in a population of 106,926 males. Eur J Ophthalmol..

[27] Zhang J (2011). Shared genetic determinants of axial length and height in children: the Guangzhou twin eye study. Arch Ophthalmol..

[28] Wong TY, Foster PJ, Johnson GJ, Klein BE, Seah SK (2001). The relationship between ocular dimensions and refraction with adult stature: the Tanjong Pagar Survey. Invest Ophthalmol Vis Sci..

[29] McMahon Gl (2009). Season of birth, daylight hours at birth, and high myopia. Ophthalmology..

[30] Wang Y (2015). Exposure to sunlight reduces the risk of myopia in rhesus monkeys. PLoS One..

[31] Guo Y (2013). Outdoor activity and myopia among primary students in rural and urban regions of Beijing. Ophthalmology..

[32] Pan CW, Ramamurthy D, Saw SM (2012). Worldwide prevalence and risk factors for myopia. Ophthalmic Physiol Opt..

[33] Koh V (2014). Differences in prevalence of refractive errors in young Asian males in Singapore between 1996-1997 and 2009-2010. Ophthalmic Epidemiol..

[34] Mandel Y (2008). Season of birth, natural light, and myopia. Ophthalmology..

[35] Norton TT, Siegwart JT (2013). Light levels, refractive development, and myopia - a speculative review. Exp Eye Res..

[36] Vannas AE (2003). Myopia and natural lighting extremes: Risk factors in Finnish army conscripts. Acta Ophthalmol Scand..

[37] Wong TY (2000). Prevalence and risk factors for refractive errors in adult Chinese in Singapore. Invest Ophthalmol Vis Sci..

[38] Smith EL, Bradley DV, Fernandes A, Boothe RG (1999). Form deprivation myopia in adolescent monkeys. Optom Vis Sci..

[39] Choh V, Lew MY, Nadel MW, Wildsoet CF (2005). Effects of interchanging hyperopic defocus and form deprivation stimuli in normal and optic nerve-sectioned chicks. Vision Res..

[40] Korea Communication Commission. Statistics on wired and wireless communication service subscribers, http://www.kcc.go.kr/user.do?mode=view&page=A02060400&dc=K02060400&boardId=1030&cp=1&boardSeq=36008 (2013).

[41] Jeong, Y. C. Media Usage Behavior of 20 Generations in Smart Generation. Gwacheon: Korea Information Society Development Institute, http://www.kisdi.re.kr/kisdi/fp/kr/board/selectSingleBoard.do?cmd=selectSingleBoard&boardId=GPK_PRESS&curPage=5&seq=28277&reStep=1305799&ctx= (2013).

[42] McBrien NA, Jobling AI, Gentle A (2009). Biomechanics of the sclera in myopia: extracellular and cellular factors. Optom Vis Sci..

[43] Stojanov O, Stokic E, Sveljo O, Naumovic N (2013). The influence of retrobulbar adipose tissue volume upon intraocular pressure in obesity. Vojnosanit Pregl..

[44] Saw SM, Gazzard G, Shih-Yen EC, Chua WH (2005). Myopia and associated pathological complications. Ophthalmic Physiol Opt..

[45] Kempen JH (2004). The prevalence of refractive errors among adults in the United States, Western Europe, and Australia. Arch Ophthalmol..

[46] Fotouhi A, Morgan IG, Iribarren R, Khabazkhoob M, Hashemi H (2012). Validity of noncycloplegic refraction in the assessment of refractive errors: the Tehran Eye Study. Acta Ophthalmol..

[47] World Health Organization. Western Pacific Region, International Association for the Study of Obesity, International Obesity Task Force. *Redefining obesity and its treatment*. (WHO; 2000).

