# Supplementary material for: An epidemiological study of the risk factors associated with myopia in young adult men in Korea
Source: Sci Rep. 2018 Jan 11;8:511. doi: 10.1038/s41598-017-18926-2 (PMC5764954; doi:10.1038/s41598-017-18926-2)
Supplement: Supplementary file 1 — Supplementary Tables [file 41598_2017_18926_MOESM1_ESM.pdf]

An epidemiological study of the risk factors associated with myopia in young adult men in Korea

Dong Cheol Lee, Se Youp Lee, Yu Cheol Kim

Supplementary Table S1. Demographic Characteristics of All Participants Over a 5-Year Period (2009–2013)

Characteristics		2009	2010	2011	2012	2013
		(n = 328,627)	(n = 356,251)	(n = 370,737)	(n = 364,156)	(n = 364,848)
Birth season	Spring (%)	74,854 (22.8)	81,588 (22.9)	87,771 (23.7)	91,319 (25.1)	92,644 (25.4)
	Summer (%)	76,415 (23.3)	82,112 (23.0)	86,001 (23.2)	85,179 (23.4)	85,858 (23.5)
	Autumn (%)	78,858 (24.0)	91,314 (25.6)	93,785 (25.3)	88,640 (24.3)	90,166 (24.7)
	Winter (%)	98,500 (30.0)	101,238 (28.4)	103,180 (27.8)	99,018 (27.2)	96,180 (26.4)
Education level	High school education or less (%)	86,467 (26.3)	102,305 (28.7)	118,396 (31.9)	119,327 (35.5)	139,107 (38.1)
	2- to 3-year college education (%)	79,810 (24.3)	85,943 (24.1)	89,836 (24.2)	82,114 (22.5)	76,377 (20.9)
	4- or 6-year university education or more (%)	162,350 (49.4)	168,003 (47.2)	162,505 (43.8)	152,715 (41.9)	149,364 (40.9)
Height (cm)	≤170 (%)	92,289 (28.1)	100,410 (28.2)	101,808 (27.5)	104,484 (28.7)	105,720 (29.0)
	171–174 (%)	89,176 (27.1)	97,285 (27.3)	101,332 (27.3)	99,625 (27.4)	100,450 (27.5)
	175–178 (%)	79,772 (24.3)	86,073 (24.2)	90,482 (24.4)	87,218 (24.0)	67,947 (18.6)
	≥179 (%)	67,390 (20.5)	72,484 (20.3)	77,115 (20.8)	72,829 (20.0)	90,731 (24.9)
	Mean ± SD	173.83 ± 5.73	173.80 ± 5.72	173.90 ± 5.72	173.73 ± 5.73	173.67 ± 5.75
Weight (kg)	≤60 (%)	90,614 (27.6)	98,239 (27.6)	103,151 (27.8)	103,035 (28.3)	103,085 (28.3)
	61–66 (%)	75,422 (23.0)	82,193 (23.1)	85,055 (22.9)	81,818 (22.5)	80,335 (22.0)

	67–75 (%)	86,634 (26.4)	93,888 (26.4)	96,798 (26.1)	94,160 (25.9)	92,764 (25.4)
	≥76 (%)	75,957 (23.1)	81,931 (23.0)	85,733 (23.1)	85,143 (23.4)	88,664 (24.3)
	Mean ± SD	68.46 ± 12.70	68.44 ± 12.71	68.43 ± 12.76	68.42 ± 12.90	68.67 ± 13.24
BMI (kg/m^2)	<18.5 (%)	32,743 (10.0)	35,179 (9.9)	37,255 (10.0)	37,442 (10.3)	38,441 (10.5)
	18.5–22.99 (%)	171,267 (52.1)	186,604 (52.4)	194,888 (52.6)	187,660 (51.5)	183,393 (50.3)
	23.00–24.99 (%)	53,921 (16.4)	58,304 (16.4)	59,774 (16.1)	59,492 (16.3)	59,173 (16.2)
	≥25.0 (%)	70,696 (21.5)	76,164 (21.4)	78,820 (21.3)	79,562 (21.8)	83,841 (23.0)
	Mean ± SD	22.63 ± 3.87	22.63 ± 3.88	22.60 ± 3.88	22.64 ± 3.92	22.73 ± 4.03
Age (years)	Mean ± SD	19.02 ± 0.45	19.02 ± 0.46	19.02 ± 0.46	19.02 ± 0.44	19.03 ± 0.43

BMI = body mass index; SD = standard deviation.

Supplementary Table S2. Prevalence of Myopia According to Possible Risk Factors

Risk factor		Prevalence of myopia (%)									
		2009		2010		2011		2012		2013	
		% (n)	*P*-value**	% (n)	*P*-value**	% (n)	*P*-value**	% (n)	*P*-value**	% (n)	*P*-value**
Birth season	Spring	51.2* (38,315)	<0.001	54.2* (44,225)	<0.001	51.5* (45,211)	<0.001	51.9* (47,388)	<0.001	52.0* (48,177)	<0.01
	Summer	51.3* (39,165)		52.8 (43,379)		50.9* (43,764)		51.2 (43,605)		51.5* (44,250)	
	Autumn	49.9 (39,345)		52.9 (48,326)		49.9 (46,784)		51.2 (45,421)		51.3 (46,291)	
	Winter	51.0* (50,212)		52.2 (52,831)		50.1 (51,732)		50.7 (50,236)		51.1 (49,179)	
Education level	High school education or less	47.8 (41,351)	<0.001	51.8 (52,961)	<0.001	47.3 (56,045)	<0.001	47.9 (61,888)	<0.001	48.3 (67,189)	<0.001
	2- to 3-year college education	40.4 (32,204)		43.3 (37,195)		42.3 (38,007)		44.2 (36,293)		45.5 (34,770)	
	4- or 6-year university education or more	57.6* (93,482)		58.7* (98,605)		57.5* (93,439)		57.9* (88,469)		57.5* (85,938)	

Height (cm)	≤170	50.3	<0.01	52.6	<0.05	50.7*	<0.05	51.3	>0.05	51.7*	<0.01
		(46,451)		(52,794)		(51,620)		(53,583)		(54,706)	
	171–174	50.8		53.1*		50.8*		51.5		51.7*	
		(45,261)		(51,679)		(51,521)		(51,261)		(51,968)	
	175–178	51.2*		53.1*		50.4		51.3		51.0	
		(40,815)		(45,727)		(45,639)		(44,744)		(34,658)	
	≥179	51.2*		53.2*		50.2		50.9		51.3	
		(34,510)		(38,561)		(38,711)		(37,062)		(46,565)	
Weight (kg)	≤60	51.6*	<0.001	53.7*	<0.001	51.9*	<0.001	53.0*	<0.001	53.4*	
		(46,788)		(52,738)		(53,498)		(54,625)		(55,096)	
	61–66	50.9*		53.3*		50.7*		51.5*		51.3	
		(38,399)		(43,828)		(43,117)		(42,099)		(41,226)	
	67–75	50.5		52.8		50.2		50.3		50.6	
		(43,790)		(49,576)		(48,575)		(47,370)		(46,918)	
	≥76	50.1		52.0		49.3		50.0		50.4	
		(38,060)		(42,619)		(42,301)		(42,556)		(44,657)	
BMI	<18.5	52.7*	<0.001	54.9*	<0.001	53.6*	<0.001	55.1*	<0.001	55.6*	
(kg/m^2)		(17,267)		(19,321)		(19,979)		(20,625)		(21,357)	
	18.5–22.99	50.9*		53.1*		50.6*		51.3*		51.4	

	(87,116)	(99,177)	(98,570)	(96,242)	(94,255)
23.00–24.99	50.4	52.5	49.8	50.2	50.6
	(27,200)	(30,583)	(29,780)	(29,880)	(61,673)
≥25.0	50.1	52.1	49.7	50.2	50.4
	(35,454)	(39,680)	(39,162)	(39,903)	(10,612)

BMI = body mass index.

* Statistically significant.

** Analysed by Pearson’s significance ratio.

Supplementary Table S3. Multivariable Logistic Regression for the Identification of Myopia Risk Factors (birth season, education level, height, and weight) and Univariate Logistic Regression for BMI

Risk factor		Multivariable (myopia)				
		2009	2010	2011	2012	2013
		OR (95% CI)	OR (95% CI)	OR (95% CI)	OR (95% CI)	OR (95% CI)
Birth season	Spring	ref*	ref*	ref*	ref*	ref*
	Summer	1.003 (0.983–1.024)	0.949 (0.931–0.968)*	0.975 (0.957–0.994)*	0.973 (0.954–0.991)*	0.983 (0.965–1.002)
	Autumn	0.952 (0.933–0.971)*	0.952 (0.934–0.970)*	0.936 (0.919–0.954)*	0.972 (0.954–0.990)*	0.973 (0.955–0.991)*
	Winter	0.986 (0.967–1.005)	0.917 (0.900–0.934)*	0.927 (0.910–0.944)*	0.937 (0.921–0.955)*	0.950 (0.933–0.967)*
Education level	High school education or less	ref*	ref*	ref*	ref*	ref*
	2- to 3-year college education	0.738 (0.724–0.752)*	0.713 (0.700–0.727)*	0.818 (0.804–0.832)*	0.865 (0.850–0.881)*	0.897 (0.881–0.913)*
	4- or 6-year university education or more	1.484 (1.459–1.509)*	1.331 (1.310–1.352)*	1.514 (1.492–1.538)*	1.509 (1.487–1.532)*	1.459 (1.438–1.481)*
Height (cm)	≤170	ref*	ref*	ref	ref*	ref*
	171–174	1.017 (0.998–1.037)	1.025 (1.007–1.044)*	1.015 (0.997–1.033)	1.022 (1.004–1.040)*	1.017 (0.999–1.035)
	175–178	1.045 (1.024–1.066)*	1.036 (1.016–1.055)*	1.005 (0.987–1.024)	1.029 (1.010–1.048)*	0.999 (0.980–1.019)
	≥179	1.051 (1.029–1.074)*	1.044 (1.023–1.066)*	1.008 (0.988–1.028)	1.026 (1.005–1.047)*	1.025 (1.006–1.045)*
Weight (kg)	≤60	ref*	ref*	ref*	ref*	ref*

	61–66	0.934 (0.916–0.953)*	0.953 (0.935–0.972)*	0.926 (0.909–0.944)*	0.914 (0.897–0.931)*	0.897 (0.901–0.935)*
	67–75	0.911 (0.893–0.929)*	0.924 (0.906–0.941)*	0.902 (0.886–0.919)*	0.868 (0.852–0.884)*	0.867 (0.876–0.907)*
	≥76	0.911 (0.892–0.930)*	0.911 (0.893–0.930)*	0.893 (0.876–0.910)*	0.873 (0.856–0.890)*	0.875 (0.868–0.900)*
BMI (kg/m^2)	<18.5	ref*	ref*	ref*	ref*	ref*
	18.5–22.99	0.928 (0.906–0.950)*	0.931 (0.910–0.953)*	0.885 (0.865–0.905)*	0.858 (0.839–0.878)*	0.846 (0.827–0.865)*
	23.00–24.99	0.912 (0.888–0.938)*	0.906 (0.882–0.930)*	0.859 (0.837–0.881)*	0.823 (0.802–0.844)*	0.818 (0.797–0.839)*
	≥25.0	0.902 (0.787–0.926)*	0.893 (0.870–0.916)*	0.854 (0.833–0.875)*	0.820 (0.800–0.841)*	0.817 (0.798–0.837)*

BMI = body mass index; CI = confidence interval; OR = odds ratio; ref = reference for OR calculation.

*Statistically significant.

Supplementary Table S4. Prevalence of High Myopia According to Possible Risk Factors

Risk factor		Prevalence of high myopia (%)									
		2009		2010		2011		2012		2013	
		% (n)	*P*-value**	% (n)	*P*-value**	% (n)	*P*-value**	% (n)	*P*-value**	% (n)	*P*-value**
Birth season	Spring	11.5* (8,638)	<0.001	12.2* (9,914)	<0.05	12.1* (10,618)	<0.001	12.8* (11,649)	<0.05	13.2* (12,213)	<0.05
	Summer	11.5* (8,783)		11.8 (9,665)		11.7* (10,097)		12.6* (10,746)		13.0* (11,163)	
	Autumn	10.8 (8,490)		11.7 (10,646)		11.4 (10,714)		12.7* (11,243)		12.8 (11,556)	
	Winter	11.4* (11,206)		11.8 (11,951)		11.6 (11,994)		12.3 (12,202)		12.8 (12,292)	
Education level	High school education or less	10.6 (9,148)	<0.001	13.9 (11,789)	<0.001	10.8 (12,831)	<0.001	11.6 (14,992)	<0.001	11.9 (16,485)	<0.001
	2- to 3-year college education	7.6 (6,076)		8.1 (6,994)		8.5 (7,616)		9.5 (7,821)		10.3 (7,844)	
	4- or 6-year university education or more	13.5* (21,893)		11.5* (23,393)		14.1* (22,976)		15.1* (23,027)		15.3* (22,895)	

Height (cm)	≤170	11.1 (10,246)	<0.001	11.4 (11,458)	<0.001	11.4 (11,637)	<0.001	12.2 (12,752)	<0.001	12.5 (13,247)	<0.001
	171–174	11.0 (9,833)		11.8 (11,471)		11.6 (11,778)		12.5 (12,434)		12.8 (12,906)	
	175–178	11.4* (9,113)		12.0* (10,292)		11.8* (10,648)		12.7* (11,083)		13.1* (8,923)	
	≥179	11.8* (7,925)		12.4* (8,955)		12.1* (9,360)		13.1* (9,571)		13.4* (12,148)	
Weight (kg)	≤60	11.3 (10,233)	>0.05	11.8 (11,597)	>0.05	11.9* (12,298)	<0.05	12.9* (13,298)	<0.001	13.1* (13,493)	<0.001
	61–66	11.1 (8,361)		11.7 (9,652)		11.5 (9,812)		12.3 (10,069)		12.7 (10,168)	
	67–75	11.3 (9,822)		11.7 (11,009)		11.6 (11,242)		12.2 (11,526)		12.6 (11,723)	
	≥76	11.5 (8,701)		12.1 (9,918)		11.7* (10,071)		12.9* (10,947)		13.4* (11,840)	
BMI (kg/m^2)	<18.5	11.9* (3,907)	<0.001	12.6* (4,419)	<0.001	12.8* (4,769)	<0.001	13.8* (5,184)	<0.001	14.2* (5,447)	<0.001

18.5–22.99	11.1	11.6	11.6	12.4	12.6
	(19,065)	(21,698)	(22,552)	(23,183)	(23,125)
23.00–24.99	11.3*	11.8	11.6	12.4	13.0
	(6,103)	(6,853)	(6,938)	(7,370)	(15,819)
≥25.0	11.4*	12.1*	11.6	12.7*	13.5*
	(8,042)	(9,206)	(9,164)	(10,103)	(2,833)

BMI = body mass index.

* Statistically significant.

** Analysed with Pearson's significance ratio.

Supplementary Table S5. Multivariable Logistic Regression for the Identification of High Myopia Risk Factors (birth season, education level, height, and weight) and Univariate Logistic Regression for BMI

Risk factor		Multivariable (high myopia)				
		2009	2010	2011	2012	2013
		OR (95% CI)	OR (95% CI)	OR (95% CI)	OR (95% CI)	OR (95% CI)
Birth season	Spring	ref*	ref*	ref*	ref*	ref*
	Summer	0.996 (0.965–1.028)	0.968 (0.940–0.998)*	0.967 (0.939–0.996)*	0.988 (0.961–1.016)	0.986 (0.959–1.014)
	Autumn	0.928 (0.899–0.958)*	0.958 (0.931–0.987)*	0.939 (0.913–0.966)*	0.994 (0.966–1.022)	0.970 (0.944–0.997)*
	Winter	0.983 (0.954–1.013)	0.966 (0.939–0.994)*	0.944 (0.918–0.971)*	0.951 (0.926–0.978)*	0.955 (0.930–0.981)*
Education level	High school education or less	ref*	ref*	ref*	ref*	ref*
	2- to 3-year college education	0.696 (0.673–0.720)*	0.681 (0.660–0.703)*	0.764 (0.741–0.787)*	0.805 (0.780–0.829)*	0.853 (0.829–0.878)*
	4- or 6-year university education or more	1.318 (1.284–1.353)*	1.245 (1.215–1.275)*	1.361 (1.329–1.392)*	1.362 (1.332–1.392)*	1.353 (1.324–1.383)*
Height (cm)	≤170	ref*	ref*	ref*	ref*	ref*
	171–174	0.984 (0.964–1.022)	1.033 (1.005–1.063)*	1.022 (0.995–1.051)	1.032 (1.004–1.060)*	1.031 (1.005–1.059)*
	175–178	1.026 (0.995–1.059)	1.053 (1.023–1.085)*	1.039 (1.009–1.070)*	1.058 (1.028–1.088)*	1.059 (1.028–1.091)*
	≥179	1.055 (1.020–1.091)*	1.087 (1.053–1.122)*	1.080 (1.047–1.114)*	1.100 (1.067–1.133)*	1.080 (1.050–1.111)*
Weight (kg)	≤60	ref*	ref*	ref*	ref*	ref*

	61–66	0.949 (0.920–0.979)*	0.960 (0.932–0.989)*	0.931 (0.905–0.958)*	0.917 (0.892–0.944)*	0.934 (0.908–0.961)*
	67–75	0.963 (0.934–0.993)*	0.945 (0.918–0.973)*	0.927 (0.901–0.954)*	0.902 (0.877–0.927)*	0.923 (0.898–0.949)*
	≥76	0.985 (0.954–1.018)	0.991 (0.962–1.022)	0.951 (0.923–0.980)*	0.961 (0.933–0.989)*	0.993 (0.966–1.022)
BMI (kg/m^2)	<18.5	ref*	ref*	ref*	ref*	ref*
	18.5–22.99	0.925 (0.891–0.959)*	0.916 (0.885–0.948)*	0.891 (0.862–0.922)*	0.877 (0.849–0.906)*	0.874 (0.847–0.902)*
	23.00–24.99	0.942 (0.903–0.983)*	0.927 (0.890–0.965)*	0.894 (0.860–0.930)*	0.880 (0.847–0.914)*	0.880 (0.848–0.914)*
	≥25.0	0.947 (0.910–0.987)*	0.957 (0.921–0.994)*	0.896 (0.863–0.930)*	0.905 (0.873–0.938)*	0.928 (0.897–0.961)*

BMI = body mass index; CI = confidence interval; OR = odds ratio; ref = reference for OR calculation.

* Statistically significant.
